# Market concentration of the Brazilian hospital medical supplementary health system

**DOI:** 10.1186/s12913-024-11280-w

**Published:** 2024-10-16

**Authors:** June Alisson Westarb Cruz, Arivelton Loeschke Gomide, Felipe Francisco Tuon, Alex Sandro Quadros Weymer, Janice Alexandra da Costa Manuel

**Affiliations:** 1 Pontifícia Universidade Católica do Paraná, Curitiba, Brazil; 2 School of Business, Pontifícia Universidade Católica do Paraná, Curitiba, Brazil; 3 Instituto Superior Politécnica de Administração e Finanças, Av. Comandante Loy, Edíficio A - Academia BAI, Entrada A, R/C Morro Bento, Luanda, Angola

**Keywords:** Supplementary health in Brazil, Market concentration, Health management, Mergers and acquisitions, Organizations

## Abstract

**Background:**

The Brazilian supplementary health market has undergone transformations in recent years due to constant mergers and acquisitions of by large corporations, contributing to the increase in market concentration, especially in the poorest and least developed regions of the country. Thus, given the care it provides and its economic relevance, understanding the fundamentals of these movements, the likely consequences and trends for the health market are relevant, important, and strategic.

**Objective:**

To understand the general and specific context of Brazilian supplementary health, its scenarios, and trends, with emphasis on the analysis of market concentration and recent mergers and acquisitions.

**Methodology:**

The research is applied, descriptive and exploratory and uses secondary data from various sources, submitted to quantitative data analysis methods. The data are organized into three groups: historical and regulatory documents; industry data; and market.

**Results:**

The results show the growing concentration of the market promoted by large publicly traded corporations, the growing relevance of tech startups on the healthcare landscape, the predominant use of the relative valuation model, with implicit multiples for asset pricing and the prevalence of corporate health plans.

**Conclusion:**

The growing concentration of the system projects a market with fewer options and less competitiveness, since the growth of large operators is evident, in addition to the relevant increase in the number of complaints from users of the system, which signals the growing gap between the expectations of users and the levels of quality care offered. The study also highlights the predominance of corporate health plans, revealing the direct relationship between access to supplementary health services and employability rates. The analysis of M&A operations, in addition to the increase in market concentration, reveals the prevalence of the use of the relative valuation model and implicit multiples for the pricing of traded assets. This denotes the future expectation of wealth generation, at least equivalent to the historical series of the sector, on the part of investors, whose frustration may signal the decreasing attractiveness of resources and M&A operations in the sector in the coming years.

## Introduction

The Brazilian health system is a complex environment that contains a blend of economic, social and political aspects and factors, combining market and public power elements with the objective of providing adequate and quality health care for the entire population of the company.

Among the numerous challenges involved in achieving this bold objective the continental dimensions of Brazil may be mentioned, as the country’s territory stretches to over 8.5 million square kilometers, with a population of approximately 215 million people [1]. Another factor to consider is the inequality of the nation’s regions at the social, structural and developmental level.

The system employs approximately 4.7 million people [2] and accounts for around 9.23% of the country’s GDP [3], with 57% of private origin and 43% of public origin [3, 4]. The country is served by the performance of the state, through the Unified Health System, and the private initiative, which operates through the so-called supplementary health system [5]. This includes a structure with 690 operators of hospital medical health plans [6], 50.5 million beneficiaries and annual revenues of 34.7 billion dollars, a context in which about 43% of Brazilian users of the system evaluate healthcare as poor or very poor, and only 9% as good or excellent [7].

The present study continues the research of Cruz et al. (2022), it presents as a research probe: What is the general and specific context of Brazilian supplementary health, its scenarios and trends, with emphasis on the analysis of market concentration and mergers and acquisitions? Now covering a longer period (from 2018 to 2022) and presents the typology of valuation methods most widely used by the health market in Brazil. The research is characterized as descriptive and exploratory, using secondary data from various sources, submitted to quantitative data analysis methods. The objective is to describe the main historical facts of supplementary health in Brazil, to map and analyze the relevant and recent movements of mergers and acquisitions that have transformed the market, as well as their main effects, actors and the main valuation approaches [8] used for the pricing of traded companies. The work also seeks to shed light on the main probable impacts and/or consequences both for companies providing services in the health market and for their beneficiaries, about the transformations inherent to the ownership of such companies and the evolution of market concentration levels.

## Health management in the Brazilian market

Public health is one of the most important issues that every government should be concerned with, and thus, government leaders should consider the proper allocation of resources and the performance of their investments for public health programs [9].

The literature is clear in stating that the markets for different hospital care services can be qualitatively affected in different ways after a hospital M&A event [10]. On the one hand, merger processes between hospitals present high potential benefits for patients, employees and managers, either in the form of quality or safety improvements for the patient or contributing to the sustainability of the system and increase the levels of satisfaction of teams [11]. On the other hand, however, recent studies have concluded that a process of this nature can culminate in a decrease in costs, efficiency gains and an increase in economies of scale, but also in increased prices, because of increased market concentration [10]. Thus, we highlight the possible occurrence of lower levels of competitiveness and the stagnation of the quality of a service over time [10].

**Fig. 1:**
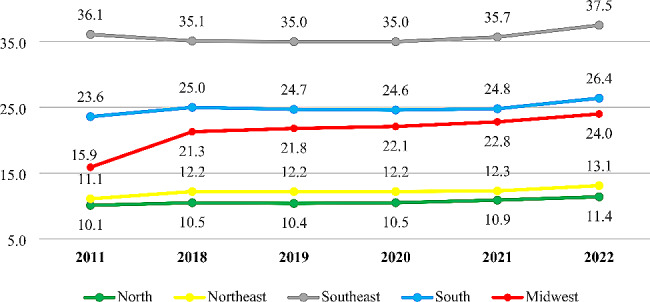
Coverage rate of supplementary health care in the different regions of Brazil. Source: Tabnet/ANS System, 2022

A point of fundamental importance for ensuring the quality of the services provided is the adoption of regionalized criteria to evaluate market concentration [12] and the population’s access [13] to services. Studies have identified impacts on the quality of care in regions with lower competitiveness among active health plans [12]. In Brazil, according to ANS data, in December 2022, medical-hospital health plans had a coverage rate of 26% at the national level. However, this coverage is not evenly distributed among the regions of the country [14], and since health plans are services used within a certain geographical delimitation, the concentration and its effects should also be analyzed from a regionalized perspective [12]. Figure 1, shows the distribution of health plan coverage rates in the regions of the country from 2011 to 2022.

The distribution of coverage rates of supplementary health services shows the great gap in equality between the regions of Brazil, especially between the North Region, which has the lowest levels of access to the service, with coverage rates close to 11.4%, and the Southeast Region, which includes the highest coverage rates in the country, around 3.3 times higher than the North.

Therefore, the expansion of investments in the health sector and the strengthening of economic, technological, industrial and social policies, as well as the regulatory frameworks that affect the production and valorization of health technologies and services, are essential [15, 16]. Consequently, a broad understanding of public health is necessary, so that policymakers can improve their management strategies and increase the quality of health for their society [9]. Regulatory policies are also expected to help to reduce the prices of hospital care and contribute to greater access to services for the population [10].

## Hospital medical supplementary health

The Brazilian health sector has developed significantly since the 1990s, with many efforts having been made through public and private means. However, Brazil continues to face many challenges in the field of health, especially due to the important socioeconomic and regional inequalities existing in the country [15].

The complexity of the industry drives healthcare organizations to deal with a rich ecosystem comprising many stakeholders, such as policymakers, funders, competitors, the public, employees, and managers [16].

The Brazilian Constitution of 1988 [17] states that every citizen has the right to enjoy quality health services. However Public Policies and government initiatives have proved insufficient and inefficient in the face of the high demands of by society. There is pressure to improve the quality of healthcare systems in such a way that even private systems also end up being questioned and pressured concerning their ability to provide quality care to the population. Even so, the Brazilian supplementary health sector has shown that access has become more limited, with the consequent exclusion of citizens due to the high costs of treatment [18].

The National Agency for Supplementary Health (ANS [6]), which was established by Law 9.961/2000 [19], is the body responsible for promoting the defense of the public interest in supplementary health care, as well as for exercising control, supervision and standardization of the private health sector to which all acts of hospitals, clinics and health plan operators are subjected. It is incumbent upon the ANS to deliberate on the processes involving the spin-off, merger, incorporation, alteration or transfer of corporate control of companies operating in the private health care market [19]. In Brazil, since the beginning of 2020, with the advent of the economic and public health crisis caused by the Coronavirus pandemic, contrary to market expectations, the demand for health plans has grown [12], a trend that has led researchers to classify Brazilian supplementary health as an expanding market [18]. In this context, special emphasis is laid to the concentration of the market in the sector through mergers and acquisitions (M&A) operations, as well as to the valuation criteria and techniques [8] used for pricing and valuation of the companies involved in each negotiation process.

## Market concentration

One of the relevant transformations suffered by the markets of hospital and medical organizations is that they have become increasingly concentrated [20]. The market share represents the percentage that each company has in the market in which it operates. The more concentrated, the greater the market share of the largest companies. Market concentration can measure to what extent a company represents of the total market, compare its competitors, identify market trends, and define growth strategies [21]. Operators seek to maximize their beneficiary bases to mitigate risks and increase their earning capacity and economies of scale [14].

Cruz et al. (2022) [22] observed that the size and market concentration of supplementary health plan operators (HPO) in Brazil have undergone important changes in recent years, with many dynamic and constant mergers and acquisitions of companies, significantly transforming the scenario. Studies have indicated the existence of a trend of reduction in the number of health plan operators in the Brazilian market and a high market share of the leading companies in the market, thus revealing a concentrated sector with oligopolies in most of the relevant markets [23].

In Brazil, there has been a gradual reduction in the number of active HPO in the country, from 745 in 2018 to 690 in 2022. It is important to highlight that consolidations can generate potential benefits for companies, such as increased operational and financial efficiency, with cost reduction, gains in scale, increased purchasing power and greater possibilities of acquiring and/or sharing high-cost technologies. Nevertheless, researchers and regulators have voiced concern regarding the risk of price increases and reduced quality services over time due to lower levels of competition [10, 11, 20, 24, 25], and this quality has already been perceived as having deteriorated, since four out of ten Brazilians disapprove of health in the country today [7].

The Herfindahl-Hirschman Index (HHI) can be used to calculate the degree of concentration of markets [12, 26]. A positive variation in the HHI, that is, an increase in the concentration of this relevant market, can lead to an increase in the number of complaints from users, and consequently, to a decrease in the quality of care [21]. However, the literature has also reported that the consolidation of health companies can generate efficiency in some circumstances and that some of these gains can be passed on to consumers [10]. Even so, although merging companies generally generate cost savings and can exploit internal synergies, with possible price reductions, benefitting both companies and consumers, the most common finding in the literature is that prices increase after the merger, even after the capture of cost reductions [25].

Thus, it falls to us to highlight the strategic importance of monitoring competition in the market and implementing policies that help increase this competition, playing a fundamental role in reducing costs and improving the quality of healthcare for users [20]. Regulatory bodies should therefore be judicious when analyzing and approving proposed mergers and adopt measures to maintain competition [24]. Such mergers should be examined by the spheres of government to assess whether the net results of these processes contribute to competitiveness in the market [20]. It should also be noted that the economic scenario of crisis is a driver of merger and acquisition movements, which leads us to expect a continuity in the trend of increasing M&A processes in the health sector [11].

## Methodology

This research is descriptive and exploratory, using secondary data from different sources submitted to quantitative data analysis methods. The history of supplementary health in Brazil as well as its main actors and M&A operations are the research object.

The research data are organized into three groups, each with its own collection and analysis approach, as shown in Table 1.

**Table 1:** General framework of research methods

Group	Data Source	Data Type	Form of Analysis
Historical and Regulatory Documents	Reference sites and surveys	Documents and laws	Descriptive document analysis
Industry Data	Industry data repository sites	Quantitative	Quantitative data analysis
Market	M&A data repository sites	Documents and quantitative data	Descriptive analysis and network analysis

The first group allows the identification and analysis of the history and relevance of the supplementary health sector in the Brazilian context, as well as the valuation methods predominantly used in M&A operations in Brazil.

The second group presents a descriptive statistical analysis of the historical series of the evolution of the sector. The data originate from industry repository databases, especially from the Tabnet portal [6], the official database of the ANS, highlighting the reliability of the data used in this study. Subsequently, using the extracted data, the values of the HHI (Herfindahl-Hirschman Index) market concentration indices and the Concentration Ratio [12, 26] of the five largest [27] (RC5), adapted to the supplementary health sector, are calculated. The results obtained for the HHI and RC5 assume values between 0 (absence of market concentration) and 1 (total market concentration). The interpretation of the results is based on the adapted scale of the credit market, where estimates between 0.10 and 0.18 represent moderate concentration and those above 0.18 mean high market concentration.

The third group describes an analysis of relational networks using Ucinet software (version 6.645), in which the analysis of the patterns of interactions of mergers and acquisitions processes between health legal entities is conducted from the identification of entities that appear as buyers or sellers in the period from 2018 to 2022.

The data sources for the identification of mergers and acquisitions operations, derived from data mining on websites that specialize in M&A, investor relations pages and communication with the corporate market, or even areas of health management, total 494 sources for the period spanning January 2018 to December 2022. Regarding the choice of each of the methods used, all are justified because they are methods consolidated by the scientific community and for their effective contribution to the fulfillment of the research objectives.

## Data presentation and analysis of the results

According to ANS data, in December 2022 there were 50,493,061 beneficiaries of supplementary health in Brazil, served through 690 hospital medical operators [6], with annual revenues of 34.7 billion dollars (US$). Table 2 presents some of the main data of the sector and its comparative evolution from 2010 to 2022 in the Brazilian context.

**Table 2:** General data on the representativeness of supplementary health in Brazil – 2010; 2022

Description	2010	2022	Source
Health expenditure (% of GDP)	8.3	9.23	^3^
Proportion of private health expenditure (%)	54.2	56.97	^3^
Proportion of public health expenditure (%)	45.8	43.03	^3^
Private Health Plans Coverage Rate (%)	22.7	26	^6^
Hospital Medical Operators (Unit)	* 1,020	690	^6^
Dental Operators (Unit)	* 485	338	^6^
Supplementary Health Users	*46,025,814	50,493,061	^6^
Individual or Family Plan Users	*9,560,381	8,978,070	^6^
Enterprise plan users	*28,877,931	35,204,096	^6^
Collective plan users	*6,628,098	6,263,896	^6^
Unidentified plan users	*959,404	46,999	^6^
Direct jobs in health (people)	-	4,691,627	^2^
Direct jobs in the private health sector (people)	-	3,699,848	^2^
Direct jobs in the public health sector (people)	-	991,779	^2^
Revenue from consideration of Private Operators (US$)	$ 14,440,009,978	$ 34,713,107,959	^6^
Assistance expenses of Operators (US$)	$ 11,567,498,940	$ 30,707,753,884	^6^
Administrative expenses of Operators (US$)	$ 2,390,311,287	$ 3,779,896,383	^6^
Carrier Business Expenses (US$)	$ 449,905,996	$ 1,243,167,931	^6^
Hospital Structures (Unidades)	6,907	7,191	^4^
Hospital Beds in Brazil (Unit)	435,793	427,047	^4^
Private Hospital Beds (Unit)	295,463	263,793	^4^
Beds per thousand inhabitants	2.23	1.99	^4^

*2011

In this analysis, a decrease can be observed in the number of active HPO in Brazil, from 1,020 in 2010 to 690 in December 2022, a fact that may contribute to the increase in the levels of concentration in this market. This, consequently, led to important changes and impacts for users of the system, in addition to the reduction of about 2% in the total number of hospital beds available, both private and public. In the meantime, the total number of hospital structures grew by approximately 4% during the period in question. Other relevant factors are the increase in the proportion of health expenditures in relation to the country’s GDP and the growth in the values of the consideration revenues earned by health plan operators.

The operators relate to their beneficiaries through contracts, prominent among which are those linked to companies, that is, so-called ‘Business Collective’ plans. Table 3 also shows that this is a characteristic common to most regions of the country.

**Table 3:** Contracting profile for medical assistance – regions (2011 and 2022), Brazil (% share, contracts in thousands)

Type of Contracting	North		Northeast		Southeast		South		Midwest	
	2022	2011	2022	2011	2022	2011	2022	2011	2022	2011
Individual or Family	392	431	1,823	1,802	4,909	5,793	1,256	1,183	586	351
	21%	26%	26%	30%	16%	20%	17%	18%	16%	15%
Business Collective	1,236	1,000	4,431	3,369	21,957	18,643	5,052	4,259	2,497	1,600
	66%	60%	63%	56%	72%	64%	69%	65%	70%	67%
Collective by Membership	239	201	819	629	3,678	4,350	1,037	1,059	483	383
	13%	12%	12%	10%	12%	15%	14%	16%	14%	16%
Uninformed / Unidentified	0	33	8	213	35	568	2	88	2	42
	0%	2%	0%	4%	0%	2%	0%	1%	0%	2%
**Total**	**1**,**867**	**1**,**665**	**7**,**081**	**6**,**014**	**30**,**579**	**29**,**354**	**7**,**348**	**6**,**589**	**3**,**568**	**2**,**376**

Source: Tabnet/ANS System, 2022

The table shows the growing trend for collective plans to the detriment of individual ones, in the comparative analysis between 2011 and 2022, in all regions of Brazil. The hegemony and growth of collective business plans reinforce the strong relationship of dependence of health plans on the levels of employability in the country. In other words, this benefit has increasingly become a privilege of the members of the population who ae gainfully employed.

## Analysis of market concentration

In Brazil, in December 2022, only a fraction of approximately 2.9% of health plan operators concentrated more than half of the users of the supplementary health market [6]. According to this analysis, the 20 largest operators concentrated around 51.6% of the beneficiaries of health plans in Brazil, approximately 26 million people. The other 48.4%, about 24.4 million beneficiaries, were distributed among the remaining approximately 670 operators, which represented over 97% of the country’s HPO base [6].

Little is known or can be said about the real effects of this concentration, especially at the regional level, however, more concentrated health plan markets tend to cause a fall in levels of well-being and the quality of care provided to users [12]. Nevertheless, the increase in concentration levels is possibly associated with mergers and acquisitions movements and market consolidation [10, 12].

Regarding the number of HPO of Medical Assistance, a significant reduction is perceived over time, from a total of 1,020 active institutions in December 2011 to only 690 in December 2022 [6]. However, the volume of beneficiaries showed an opposite trend, rising from 46,025,814 in 2011 to 50,493,061 in December 2022, according to ANS data. As for the concentration indices, both the market concentration ratio (RC5) and the HHI also suffered a considerable growth variation during this period. Table 4 details the evolutionary history of the number of beneficiaries and HPO in Brazil, as well as the evolution of the RC5 and HHI market concentration indices in the period between 2011 and 2022.

**Table 4:** Evolution of beneficiary bases, HPO and concentration indices RC5 and HHI (2011 to 2022) of Brazil

Year	Total Beneficiaries	Total Hpo	RC5	HHI (Σ1–10)
2011	46,025,814	1,020	0.22	0.32
2012	47,846,092	979	0.23	0.33
2013	49,491,826	927	0.24	0.35
2014	50,531,748	892	0.27	0.37
2015	49,279,085	832	0.27	0.38
2016	47,685,266	797	0.27	0.38
2017	47,160,876	768	0.27	0.37
2018	47,142,534	745	0.28	0.38
2019	47,074,052	726	0.28	0.39
2020	47,476,332	704	0.29	0.40
2021	48,966,460	696	0.31	0.42
2022	50,493,061	690	0.31	0.42

Source: Tabnet/ANS System, 2022

It can be observed in this analysis that the reduction in the number of active HPO occurs concomitantly with the increase in the indices of market concentration ratio (RC5, concentration in the five largest) and HHI (sum of the ten largest) in the period. In other words, the level of concentration of the supplementary health market has grown consistently, as the number of beneficiaries has become increasingly concentrated among the largest operators year by year. In addition, it can be concluded that this market, because it presents RC5 indices higher than 0.18, shows high market concentration and this concentration only continues to grow.

To complement this analysis bias, Table 5, presents a horizontal analysis of the evolution of the distribution of the numbers of beneficiaries, grouping them by size in the years 2011 and 2022.

**Table 5:** Distribution of beneficiaries and horizontal analysis (2011 and 2022) of Brazil

Grouping	2011	2022	HA
Over 500,000 beneficiaries	17,600,739	24,816,108	41.0%
100,001 to 500,000 beneficiaries	12,276,731	13,190,718	7.4%
50,001 to 100,000 beneficiaries	6,410,403	5,165,473	-19.4%
20,001 to 50,000 beneficiários	5,567096	4,594,439	-17.5%
10,001 to 20,000 beneficiaries	2,482,548	1,663,316	-330%
5,001 to 10,000 beneficiaries	1,080,166	737,391	-31.7%
2,001 to 5,000 beneficiaries	451,840	252,218	-44.2%
1,001 to 2,000 beneficiaries	110,730	49,353	-55.4%
101 to 1,000 beneficiaries	44,755	23,672	-47.1%
1 to 100 beneficiaries	806	373	-53.7%
**TOTAL BENEFICIARIES**	**46**,**025**,**814**	**50**,**493**,**061**	**9.7%**

Source: Tabnet/ANS System, 2022

This analysis also allows us to observe the trend of increasing the concentration of beneficiaries by larger operators over time, demonstrated by the growth of around 41% in the number of beneficiaries linked to operators that have more than 500 thousand beneficiaries. This is concomitant with the decrease in the number of users in HPO with less than 100 thousand beneficiaries, with a greater decline among smaller operators, resulting in the obvious effect of increasing the concentration rates of this market.

The regional distribution of beneficiaries of supplementary health services shows an imbalance when compared individually with the respective populations of each region. By analyzing the coverage rate of supplementary healthcare and the RC5 index of concentration ratio by regions of Brazil, it is possible to understand, in a practical way, that the continental dimensions of the country denote extremely different regional realities. Table 6 shows the evolution of the scenario in terms of coverage and concentration.

**Table 6:** Supplementary health coverage rates, RC5 concentration ratio and regional HHI-ac10 (2011 and 2022) of Brazil

Region	Beneficiaries	Coverage		RC5		HHI (Σ1–10)	
	2022	2022	2011	2022	2011	2022	2011
North	1,867,031	11.4%	10.1%	0.60	0.52	0.73	0.65
Northeast	7,080,665	13.1%	11.1%	0.52	0.36	0.66	0.49
Southeast	30,578,954	37.5%	36.1%	0.36	0.27	0.46	0.39
South	7,347,693	26.4%	23.6%	0.27	0.26	0.40	0.40
Midwest	3,568,446	24.0%	15.9%	0.47	0.36	0.64	0.54
Brazil	50,493,061	26.0%	23.7%	0.31	0.22	0.42	0.32

Source: Tabnet/ANS System, 2022

While all regions show increased market concentration from 2011 to 2022, overall coverage rates experienced more discrete shifts during the same period. While Brazil saw its coverage grow by an average of 2.3% points, the positive highlight is for the Midwest Region, with an increase of this indicator by around 8.1% points. The high levels of inequality between the regions can also be represented by the comparison between the North Region, which has the worst rates of private coverage, with rates close to 11.4%, while the Southeast Region includes the highest coverage rates in the country, with about 37.5% of the population having access to healthcare through private institutions.

The growth in the RC5 concentration ratio was sharper in the Northeast Region, from 0.36 in 2011 to 0.52 in 2022, while the lowest growth occurred in the South, from 0.26 to 0.27, which is still the Region with the lowest RC5 concentration in the country. Meanwhile, the North Region has the highest concentration, reaching 0.60 in 2022, which implies that about 60% of the beneficiaries are concentrated in the five largest operators in this region. Similarly, the values of the HHI-ac10 (sum of the HHI of the ten largest operators) saw a positive variation in all regions, in proportions like those experienced by the RC5.

**Fig. 2:**
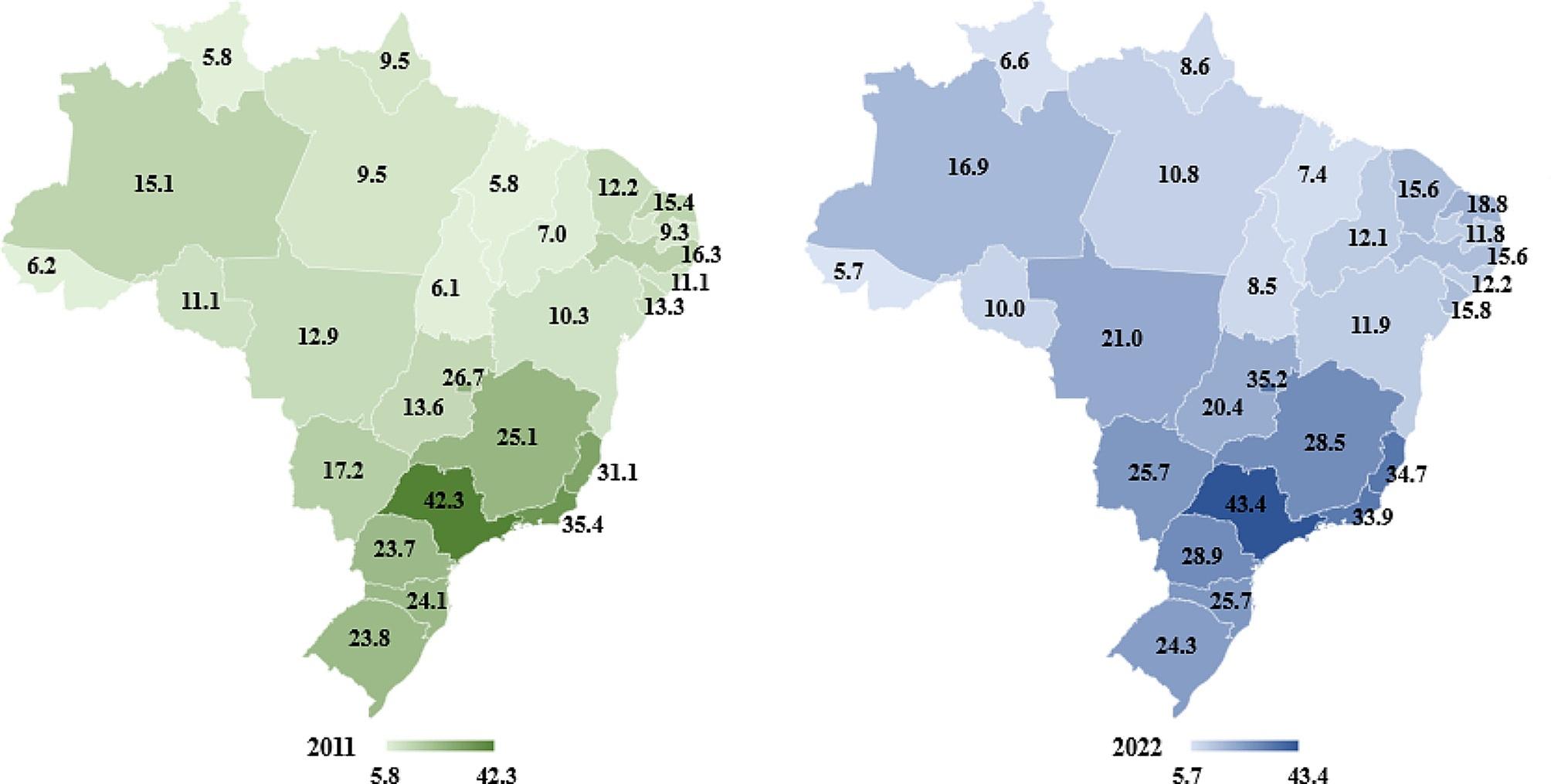
Rates (%) of private health care coverage per federation unit (2011 and 2022) of Brazil. Source: Tabnet/ANS System – Developed by the authors, 2022

The regional imbalance that was identified is even more noticeable in the analysis of private health care coverage rates federal unit, as shown in Fig. 2.

The figure presents the scenario of great inequality between the Brazilian states, in a comparison of the evolution between 2011 and 2022. For instance, the coverage in Acre and Roraima in 2022, of only 5.7% and 6.6%, respectively, contrasts significantly with states such as São Paulo and the Federal District, which offered private care to 43.4% and 35.2% of their populations in 2022, respectively. In a comparative analysis of the evolution between 2011 and 2022, it is also possible to observe both growing trends in the rates of supplementary health coverage in several states, such as São Paulo, Paraná, Minas Gerais, Goiás, and a decrease in this coverage in states such as Acre, Rondônia and Amapá, thus revealing the great differences between the states of the federation in this regard.

The evolution of the behavior of the national HHI and the number of beneficiaries of each of the ten largest HPO in the period between 2011 and 2022 is shown in Table 7.

**Table 7:** Evolution of beneficiaries and HHI of the current ten largest operators (2011 and 2022) of Brazil

Private Health Institution	Beneficiaries			HHI		
	2011	2022	AH	2011	2022	∆
Hapvida Medical Assistance	1,134,584	3,973,412	250.2%	0.025	0.079	219.2%
Bradesco Health	2,988,834	3,446,645	15.3%	0.065	0.068	5.1%
Gndi - Notre Dame Interméd.	2,140,143	3,381,349	58.0%	0.046	0.067	44.0%
Amil Medical Assistance	2,624,621	2,703,278	3.0%	0.057	0.054	-6.1%
Sul America Cia De Seguros	1,279,444	2,119,146	65.6%	0.028	0.042	51.0%
Unimed National Central	1,168,769	1,979,169	69.3%	0.025	0.039	54.4%
Unimed - Belo Horizonte	971,061	1,496,565	54.1%	0.021	0.030	40.5%
Unimed - Rio	774,619	725,856	-6.3%	0.017	0.014	-14.6%
Unimed Porto Alegre	468,638	696,773	48.7%	0.010	0.014	35.5%
Unimed Health Insurance	291,395	641,378	120.1%	0.006	0.013	100.6%

Source: Tabnet/ANS System – Developed by the authors, 2022

This analysis shows the great growth experienced by some of these companies, both in the number of beneficiaries and in their representativeness over the base (HHI), and consequently in the concentration of this market, especially in the case of Hapvida.

This company increased its number of customers by 250% and its HHI concentration by 219%, in addition to taking the lead in the number of beneficiaries among the Brazilian HPO. It is also worth highlighting the strategic relevance of these operators, which together concentrated more than 21 million beneficiaries in December 2022, according to the ANS.

## Analysis of the mergers and acquisitions market

**Fig. 3:**
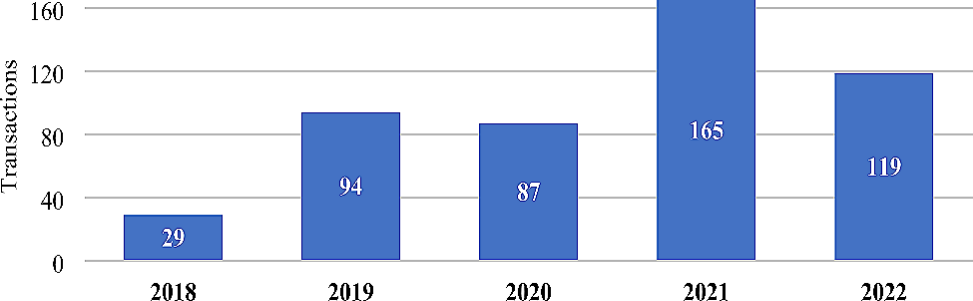
Evolution of the number of mergers and acquisitions between health companies of Brazil. Source: Repository sites for industry data, M&A and investor relations companies. Developed by the authors, 2022

The increase in the number of beneficiaries and in the market concentration index can occur in two main ways: by ordinary (or organic) growth or through mergers and acquisitions [22]. Figure 3 shows the evolution of the number of M&A transactions involving companies in the health sector in Brazil in the period between January 2018 and December 2022.

**Fig. 4:**
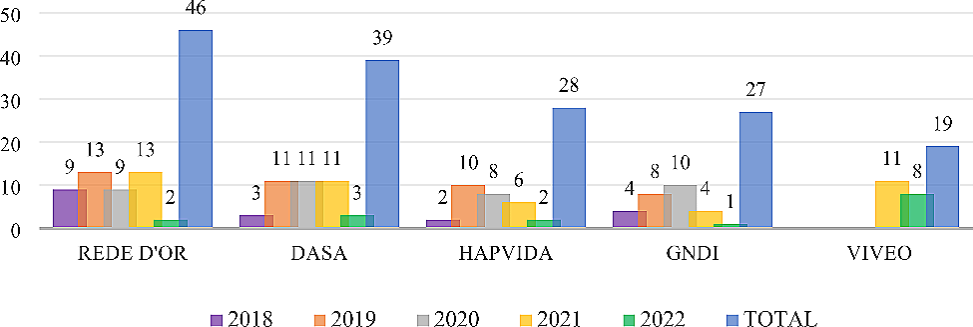
M&A activity of the five main buyer entities (2018 to 2022) of Brazil. Source: Repository sites for industry data, M&A and investor relations companies. Developed by the authors, 2022

This evolution shows a relevant growth in the number of transactions from 2019, which contributed to the increase in the size of the entities that positioned themselves as buyers of assets, and consequently, to the increase in market concentration indices (HHI and RC5) in this period. It is also observed that M&A operations are responsible for the main movements of change in the concentration and distribution of beneficiaries among the largest HPO in the country, once again highlighting the performance of the operators Hapvida and GNDI, which together with Rede D’Or, DASA and Viveo, constitute the group of the five most active companies in the Brazilian health market from 2018 to 2022, as shown in Fig. 4.

The figure shows the amounts of transactions (or M&A movements) in the period in question by the five most active companies in mergers and acquisitions, all of which are publicly traded companies in the Brazilian market.

In this context, we also highlight the relevant merger movement (business combination) between the operators Hapvida and GNDI, which resulted in the largest company in the segment in Brazil, with more than seven million beneficiaries [6] of medical-hospital care and with a projected HHI of 0.146 in relation to the beneficiary base of December 2022. In 2021, this new company had a total net revenue of around $ 4.5 billion and a total of approximately 15 million beneficiaries of hospital and dental health care, numbers that accredit it as one of the largest companies in the world in the healthcare sector. This operation was approved by CADE [28], with publication in the Official Gazette of December 16, 2021, passing to Hapvida the indirect corporate control of the health care plan operators that make up the GNDI.

Table 8, presents a summary of the ten main purchasing entities involved in M&A transactions in the healthcare sector, in addition to providing data on annual net operating revenue (ROL), type of operation, degree of general centrality in the healthcare market and number of M&A transactions carried out by these companies.

**Table 8:** Top ten purchasing entities in M&A (2018 to 2022) of Brazil

Buyer Entity	Operation Type	NOI* (US$)	Degree of Centrality	M&A Operations
Rede D’Or	Hospital	4,338,150,895	0.092	46
Dasa	Diagnosis	2,061,630,219	0.079	39
Hapvida	HPO	1,964,890,656	0.058	28
GNDI	HPO	2,501,868,787	0.058	27
Viveo	Other	1,236,333,797	0.041	19
Afya Educacional	Medical Education	348,454,871	0.041	19
Grupo Fleury	Diagnosis	684,711,928	0.032	15
Oncoclínicas	Medical Clinic	537,196,819	0.024	11
Kora Saúde	Hospital	250,894,632	0.021	10
Hermes Pardini	Diagnosis	394,790,855	0.021	10

* Net Operating Income - fiscal year 2021

Source: Repository sites for industry data, M&A and investor relations companies. Developed by the authors, 2022.

An initial identification of the diversity of the typologies of the operations is possible, where two HPO are placed. The wide classification of all the companies listed in this analysis is also noteworthy, emphasized by the highlighted ROL values. The degree of centrality presented reflects the relevance of the largest companies in the face of market consolidation movements.

2021 proved to be the year with the highest volume of operations during the period in question, with around 165 transactions. 2022 saw the continuity of the transformation of the market through M&A operations, in which the high participation level of investment groups and consortia as buying entities (or investors) may be highlighted. This process resulted in an expressive direction of operations involving technology startups, the healthtechs, which have played a leading role in the market by presenting innovative solutions in the face of the new difficulties arising from the context of the COVID-19 Pandemic. This, consequently, aroused considerable interest on the part of both investors and large established companies. Furthermore, large corporations operating in the typologies of Hospital groups (Kora Saúde, Mater Dei and Rede D’Or), Diagnostic networks (Fleury Group, DASA, Sabin Group and Hermes Pardini), helthtechs (Bionexo), Medical Education (Afya Educacional) and Medical Clinics (Oncoclínicas) maintained their strategic movements of non-organic growth.

**Fig. 5:**
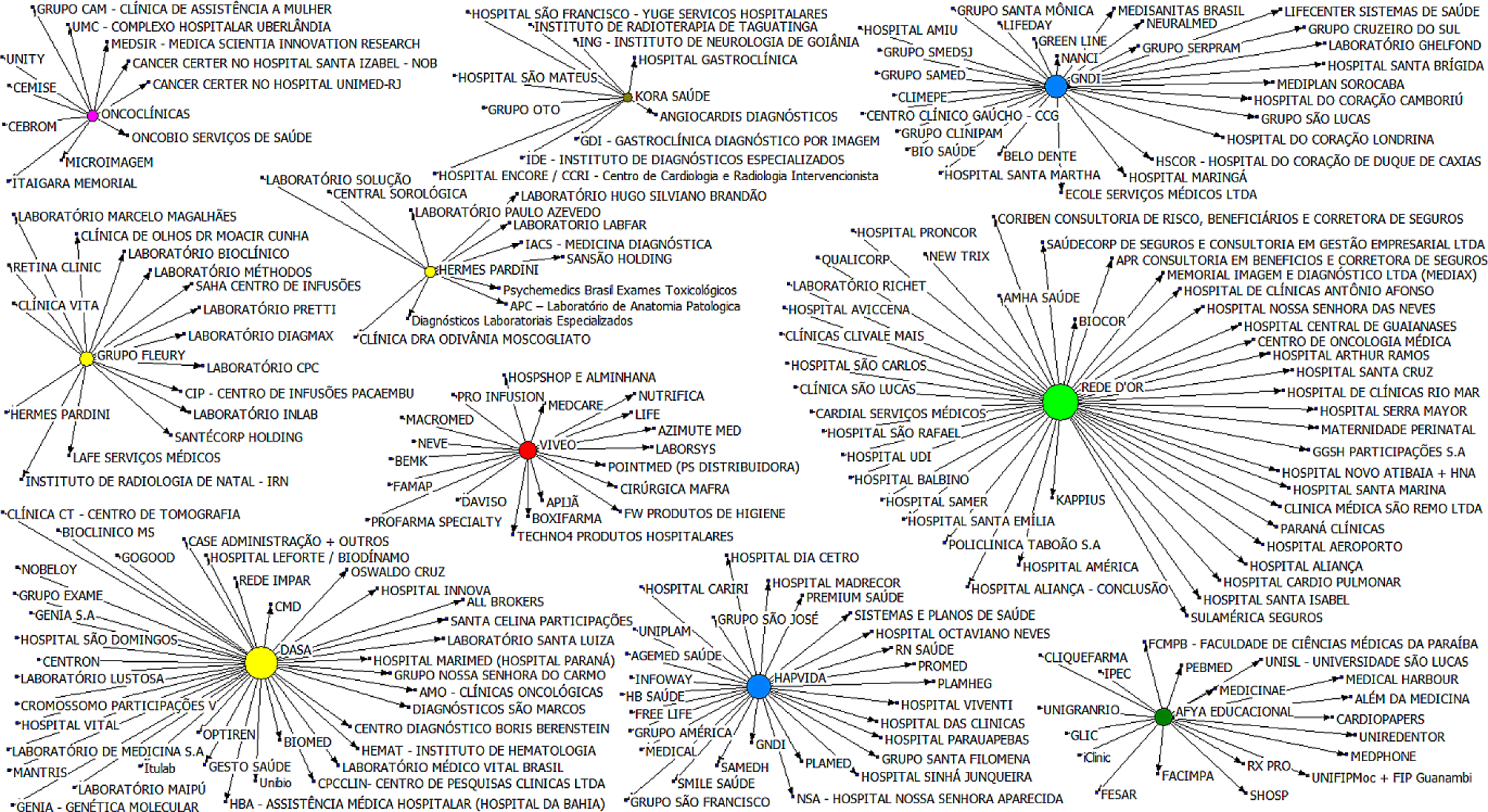
Sociogram of mergers and acquisitions – the ten largest in degree of centrality (2018 to 2022) of Brazil. Source: Repository sites for industry data, M&A and investor relations companies. Developed by the authors, 2022

In a consolidated analysis of the entire study period (2018 to 2022), among the main purchasing entities, the presence of representatives of Health Plan Operators, Hospital groups, Diagnostic networks, Medical Education, Medical Clinics, Healthtechs, Industries and Distributors can be identified, broadly covering all the links in the health chain. The following sociogram, presented in Fig. 5, illustrates the participation of the ten companies with the highest degree of centrality from 2018 to 2022 in mergers and acquisitions.

This analysis highlights the main movements that led to the current format of market consolidation, the result of non-organic growth obtained through the acquisitions of companies. We highlight the movements of the ten most active companies in M&A operations, and, consequently, with the highest degrees of centrality. Among the main purchasing entities are present representatives of Health Plan Operators, Hospital groups, Diagnostic networks, Medical Education centers, Medical Clinics, Healthtechs, Industries and Distributors, broadly covering all the links in the health chain. In addition, analyzing the scenario of the concentration of beneficiaries of health plans in the Brazilian market, the RC5 concentration index was found to have reached the level of 0.31 in 2022, which means that around 31% of the beneficiaries are concentrated in the five largest HPO in the country (Hapvida, Bradesco, GNDI, Amil and Sulamérica). Finally, it is also worth mentioning the fact that all the companies positioned among the ten largest in centrality and the five largest in RC5, are publicly traded corporations in the Brazilian market.

## Analysis by type of operations

**Fig. 6:**
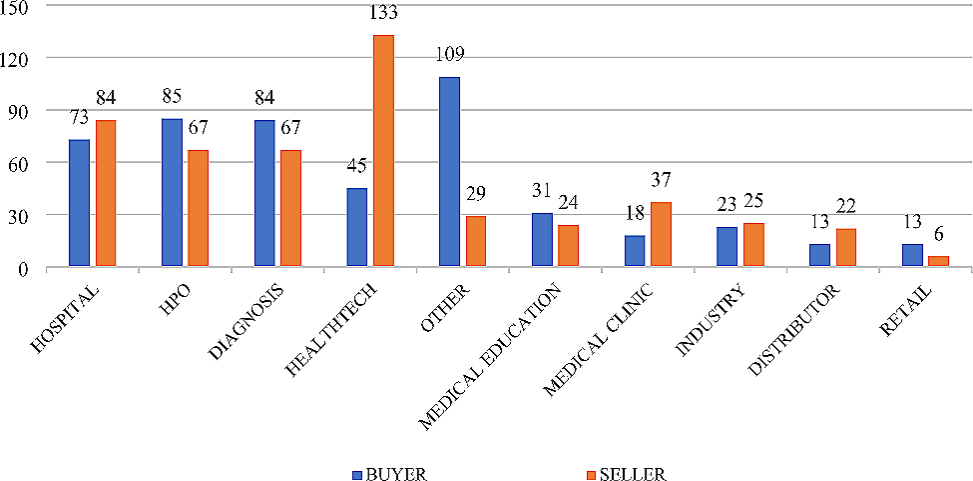
M&A transactions by typology of buyers and sellers (2018 to 2022) of Brazil. Source: Repository sites for industry data, M&A and investor relations companies. Developed by the authors, 2022

Considering the total set of operations carried out in the Brazilian health market, where 494 mergers and acquisitions transactions between January 2018 and December 2022 were mapped, and analyzing the typology of the companies involved, both as buyers and sellers, it is possible to highlight again the active participation of HPO, hospitals, diagnostic centers and, especially, the so-called health techs, technology companies focused on the health market, as shown in the following Fig. 6.

It may be highlighted that health techs are the most frequently sold type of asset, with 133 sale operations and 45 purchase operations. These companies are, in essence, startups with a technological profile focused on the development of innovative solutions that help overcome challenges in the health sector. They have experienced considerable growth and appreciation, especially since the COVID-19 Pandemic that leveraged the need for digital transformation, and consequently the commercial interest in technological solutions and tools offered by these organizations. This is reflected in the volume of M&A transactions involving this type of company. Special emphasis is placed on the group classified as ‘Other’, which figures in the analysis as the main buyer in quantitative terms, with 109 operations during the period in question. Among the main representatives of this typology are investment consortia and Venture Capital companies, which have been especially eager to acquire startups.

## Valuation approach

**Fig. 7:**
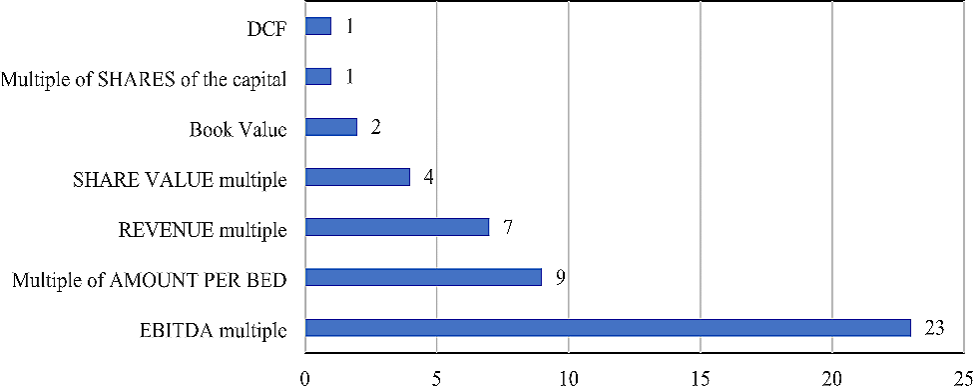
Valuation approaches used in Health M&A processes (2018 to 2022) of Brazil. Source: Repository sites for industry data, M&A and investor relations companies. Developed by the authors, 2022

Of the main valuation approaches [8] used in the pricing processes of the assets traded in mapped M&A transactions, in the universe of 494 mergers and acquisitions transactions carried out between January 2018 and December 2022 in the Brazilian healthcare market, the valuation techniques of only 47 operations were disclosed. In this respect, the methods of analysis used are presented in Fig. 7.

It was observed that 97.9% of the evaluations disclosed were theoretically based on the relative valuation model [8], that is, the one where the prices of the evaluated assets derive from prices of comparable assets traded in the market. This is done either through a direct comparison of the goods or using implicit multiples related to certain representative parts of the asset, such as the value per bed in the case of hospitals, or result indicators generated by the evaluated company, such as multiples of revenue or profit in each period. The remaining 2.1% of the valuations used the discounted cash flow (DCF) method for pricing their assets. Among the cases of relative valuation, it was observed that 43 valuations (91.5%) used the implicit multiples model, where 23 transactions (48.9%) applied the multiple of EBITDA, 9 operations (19.1%) used the multiple of value per hospital bed, 7 approaches (14.9%) were based on a multiple of revenue, 4 cases (8.5%) used a multiple of value of the share, 2 processes (4.3%) applied the book value as a reference and one approach (2.1%) was based on a multiple of shares of the company’s share capital.

This analysis, in an approximate way, demonstrates unequivocally that multiple methods are common in mergers and acquisitions in the health market. Since this practice employs comparative analysis, either of financial performance or of the value of representative parts of a given asset or assets, or even of the market value of similar companies identified in transactions that have already been made, it can thus reflect the value practices in force in the market at the time for that asset, enabling a simpler and faster evaluation process. The practice is also less liable to errors in estimating growth and future performance, which could distort the result of the evaluation since it uses a comparison with the performance and growth potentials of similar players in that context and market. The negative effect caused by the absence or scarcity of economic-financial information can also be minimized with the use of multiples, since, again, pragmatic comparisons are made regarding the structure, equipment, available physical resources, and characteristics of the consumer market in question. Moreover, the comparative analysis of the accounting practices used by companies, such as the forms of classification and accounting of assets, liabilities, expenses, and revenues, among others, also lends greater quality to the valuation process, since the similarity of such practices infers alignment and similarity of characteristics, and consequently of value, between the companies or assets under study.

## Discussion and conclusion

The Brazilian supplementary health market presents constant growth in its concentration levels, a fact reflected both in the indicators of market concentration widely analyzed and discussed in this study and in the real decrease in the number of active operators, as well as in the increase in the concentration of users in large operators. However, the exact effects, whether beneficial or detrimental to consumers, cannot be stated. Nevertheless, the detailed analysis of the data, the context of supplementary health, and the probable impacts and/or benefits for users of the system, points to the need to adopt risk mitigation measures and actions to encourage market competition to develop the sector through aspects such as cost effectiveness analysis [29–32] and humanization [31].

The figures reveal a continuous movement of growth of the large operators, institutions with a concentration of more than 500 thousand beneficiaries, which experienced an average increase of 41% in their bases from 2011 to 2022, contrasting sharply with the inverse movement that occurred in the case of the smaller operators. Another important piece of evidence of the increase in this concentration is the continuation of the aggressive increase in the RC5 indicator, from 0.22 in 2011 to 0.31 in 2022. It is also noteworthy that this scenario of concentration is greater in Brazilian regions with lower coverage rates by private operators, such as the North Region, which has coverage of 11.4% and achieved an RC5 of 0.60 in 2022. This implies that 60% of the beneficiaries are concentrated in the five largest HPO in that region, like that scenario presented by the Northeast Region. In contrast, the South and Southeast regions, the richest and most highly developed in the country, have private coverage rates of 26.4% and 37.5%, respectively, combined with RC5 concentration indices of 0.27 and 0.36, respectively. These figures suggest better conditions of competition and service provision in these regions compared with the others.

Another relevant item of data that enables a better understanding of the context and its effects, to be explored in future research, is the increase of more than 138% in the number of complaints from users of operators in the last five years, a fact that also suggests a decrease in the levels of quality and availability of the services offered.

Thus, from the broad analysis of the Brazilian scenario, and in view of the high and growing rates of market concentration and dissatisfaction of users of the system, it is reasonable to say that the levels of competitiveness and competition tend to be lower, thereby increasing the risks of reducing the quality of care and increasing prices for the final consumer.

Since the economic crisis scenario acts as a motivator and driver of mergers and acquisitions, there is an urgent need for companies to continue the constant development of synergistic mechanisms that provide financial, technological, and operational gains and contribute to the reduction of their costs and improve the quality of their services. As a result, they can become increasingly attractive to beneficiaries, attracting, and retaining them. In the process, they will remain healthy and competitive, and this will enable their growth and continuity in the market.

However, the study showed that a drive for ordinary growth alone has not been the only way to increase the degree of market share used by companies, nor the most effective. The continuing strong movements of mergers and acquisitions can also be observed. This strategy is used as a tool both for the growth of the beneficiary and customer bases and for the leverage of synergistic and operational gains that help reduce transaction costs, as well as an instrument for the consolidation of large market concentration operations.

The relevant increase in the volume of such transactions identified and analyzed in this study shows that large corporations continue to be eager and financially able to continue the great movements of formation and consolidation of the market, with special emphasis on the participation of health techs as the most purchased assets. This is related to the effect exerted by the COVID-19 Pandemic that triggered the emergence of new demands and technological solutions to meet the needs of the population and, consequently, of a vigorous and attractive niche market. This market has attracted the attention of both large players operating in the market and financial institutions and investor groups.

Finally, the present study identified that, among the pricing practices of assets traded in transactions of mergers and acquisitions, the use of market multiples (relative valuation) is the main form of valuation approach used in the Brazilian supplementary health market, with emphasis on the use of EBITDA multiples, which evidences the expectation of wealth generation by investors, whose frustration may denote the decrease in attractiveness of M&A resources and operations in the sector in the coming years.

## Data Availability

The data sets generated and / or course during the current study are available in the files sent to the journal.

## Declarations

### Experiments in humans

The research does not address experiences in humans and therefore does not require submission to institutional evaluation committees, according to the terms of the regulations of the two institutions promoting the research, of Brazilian, Angolan and international research laws, as it is a research focused on Management in the health area.
